# Understanding the Molecular Basis of Fragile X Syndrome Using Differentiated Mesenchymal Stem Cells

**DOI:** 10.22037/ijcn.v15i4.22070

**Published:** 2022-01-01

**Authors:** Zahra FAZELI, Sayyed Mohammad Hossein GHADERIAN, Hossein NAJMABADI, Mir Davood OMRANI

**Affiliations:** 1 Department of Medical Genetics, Faculty of Medicine, Shahid Beheshti University of Medical Sciences, Tehran, Iran; 2 Urogenital Stem Cell Research Center, Shahid Beheshti University of Medical Sciences, Tehran, Iran; 3 Genetics Research Center, University of Social Welfare and Rehabilitation Sciences, Tehran, Iran

**Keywords:** Fragile X Syndrome, Molecular Mechanism, Mesenchymal Stem Cells, SLITRK2, SLITRK4, MECP2, GABRA3

## Abstract

**Objectives:**

Fragile X syndrome (FXS) has been known as the most common cause of inherited intellectual disability and autism. This disease results from the loss of *fragile X mental retardation protein *expression due to the expansion of CGG repeats located on the 5’ untranslated region of the fragile X mental retardation 1 (*FMR1*) gene.

**Materials & Methods:**

In the present study, the peripheral blood-mesenchymal stem cells (PB-MSCs) of two female full mutation carriers were differentiated into neuronal cells by the suppression of bone morphogenesis pathway signaling. Then, the expression of genes adjacent to CGG repeats expansion, including SLIT and NTRK-like protein 2 (*SLITRK2*), SLIT and NTRK-like protein 4 (*SLITRK4*), methyl CpG binding protein 2 (*MECP2*), and gamma-aminobutyric acid receptor subunit alpha-3 (*GABRA3*), were evaluated in these cells using SYBR Green real-time polymerase chain reaction.

**Results:**

The obtained results indicated that the expression of *SLITRK2* and *SLITRK4* were upregulated and downregulated in the neuron-like cells differentiated from the PB-MSCs of females with *FMR1* full mutation, compared to that of the normal females, respectively. Furthermore, the expression of *MECP2* and *GABRA3* genes were observed to be related to the phenotypic differences observed in the female *FMR1* full mutation carriers.

**Conclusion:**

The observed association of expression of genes located upstream of the *FMR1* gene with phenotypic differences in the female carriers could increase the understanding of novel therapeutic targets for patients with mild symptoms of FXS and the patients affected by other *FMR1*-related disorders.

## Introduction

Fragile X syndrome (FXS, OMIM 309550) is the most common cause of inherited mental retardation that results from the expansion of CGG repeats in the 5’ untranslated region (5’ UTR) of the fragile X mental retardation 1 (*FMR1*) gene (1). The incidence of FXS has been estimated approximately at 1 in 4,000 males and 1 in 8,000 females (2). Almost 25% of boys and 6% of girls affected by FXS will develop autism spectrum disorders (ASD); however, only 1-2% of ASD patients presented FXS (3-4). Men affected by FXS usually exhibit moderate mental retardation and often have physical and behavioral characteristics (5). Females with *FMR1* gene full mutation showed intelligence quotient scores within the range of normal to less than 70 points (6). Furthermore, failure in short-term memory, executive function, and visual memory and language impairments are very common in individuals with FXS (7-10). 

A genetically-engineered mouse model for FXS did not show characteristics similar to methylation and gene silencing of the *FMR1* gene in humans (11). Epigenetic mechanisms in humans and mice are different. Therefore, the mice was not suitable models to study *FMR1* epigenetic mechanisms. Furthermore, the differences between mouse and human brain structures reflected the challenges in understanding the molecular mechanisms of abnormal brain development and function in FXS patients. Studies indicated that brain development in humans required more time than in mice. The human brain is also very dependent on interneurons and astrocytes. Clear differences were observed in the nervous system of mouse models and patients with FXS. For example, the development of human interneurons occurs over a prolonged period and requires the integration of unique mechanisms to generate numerous interneurons (12-13). The study of FXS epigenetic mechanisms in humans could provide a better understanding of the disease. 

Numerous studies have been performed on embryonic stem (ES) and induced pluripotent stem (iPS) cells derived from patients with FXS. These cells could be very beneficial to study molecular mechanisms and development stages of diseases. For example, iPS cells can be used as a model to study *FMR1* gene transcription switching from active to inactive status during differentiation. Although human iPS and ES cells were very beneficial, there were some limitations on the use of these cells. The application of ES cells isolated from human embryos was associated with ethical problems, and the successful generation of iPS cells from somatic cells was dependent on the used method (14). 

Recently, the identification and isolation of stem cells derived from different organs have provided a new cellular model to study the molecular mechanisms of different diseases. However, it is not possible to isolate stem cells from some tissues, including the central nervous system. Stem cells derived from other tissues have been known to have differentiation ability into the cells of other tissues. They could provide a new source of cells to study in vitro. Recent studies have demonstrated that mesenchymal stem cells (MSCs) can differentiate into different cell types, including neuronal cells (15). In the present study, peripheral blood-mesenchymal stem cells (PB-MSCs) were used to differentiate into neuron-like cells. Although bone marrow is the main source of MSCs, its cell separation technique is an invasive procedure. The MSCs could be isolated from peripheral blood by a non-invasive method as previously published (15). 

The product of the *FMR1* gene, *fragile X mental retardation protein* (*FMRP*), plays an important role in the regulation of the translation of the dendritic messenger ribonucleic acid (mRNA) molecules in response to the activation of synapses (16-17), indicating that the lack of this protein was associated with mental retardation in males with FXS (18). Although the molecular mechanism of FXS has been revealed to be the expansion of CGG repeats located on the 5’ UTR of the *FMR1* gene and its abnormal methylation, some studies indicated that the hyper-methylation of the *FMR1* gene could directly or indirectly affect the expression of some of its downstream genes (19). For increasing the understanding of FXS etiology, the expression of four genes adjacent to the CGG repeats expansion of the *FMR1* gene (i.e., SLIT and NTRK-like protein 2 (*SLITRK2*), SLIT and NTRK-like protein 4 (*SLITRK4*), gamma-aminobutyric acid receptor subunit alpha-3 (*GABRA3*), and methyl CpG binding protein 2 (*MECP2*)) were evaluated in the females with *FMR1* gene full mutation and different phenotypic characteristics. 

## Materials & Methods

**Patients**

In this study, 20 ml peripheral blood samples were obtained from two females with full mutation referred to Kariminejad & Najmabadi Pathology and Genetics Center, Tehran, Iran (named NF and MF, respectively), as well as a normal female with a normal allele. The expansion of CGG repeats upstream of the *FMR1* gene was determined using triplet-primed polymerase chain reaction (TP PCR), as confirmed by AmplideX FMR1 PCR kit (Asuragen, Austin, TX, USA) and Southern blotting. 

**Methylation Analysis**

The methylation pattern of CGG repeats expansion upstream of the *FMR1* gene was determined by methylation-specific polymerase chain reaction (MS PCR) followed by capillary electrophoresis. At first, sodium bisulfite modification was performed on genomic deoxyribonucleic acid (DNA) using EpiTect® Bisulfite (Qiagen, UK). Then, the modified DNA was subject to MS PCR using primers specific for methylated (Met PCR) and unmethylated PCR (non-Met PCR). The primers were as previously described (20). The PCR was prepared in a final volume of 25 μl containing 0.8 mM deoxyribonucleotide triphosphate (Qiagen, UK), 1X PCR buffer with 2.5 mM magnesium chloride, 1.5x Q-Solution (Qiagen, UK), 100 ng modified DNA, 0.4 μM of forward and reverse primers, and 2.5 U HotStarTaq DNA polymerase (Qiagen, UK). The PCR conditions included one initial denaturation at 95 °C for 10 minutes, 14 cycles (98 °C for 1 minute, 74.2 °C (Met PCR) or 68.1 °C (non-Met PCR) for 1 minute, and 72 °C for 2 minutes with 0.5 °C/s ramp rate; reducing the annealing temperature by 0.5 °C each cycle), 19 cycles (98 °C for 1 minute, 67.2 °C (Met PCR) or 61.1 °C (non-Met PCR) for 1 minute, and 72 °C for 2 minutes with 0.5 °C/s ramp rate), and final extension at 72 °C for 10 minutes. The MS PCR products were resolved by capillary electrophoresis. Then, electropherograms were analyzed with Peak Scanner Software (version 2.0 ; Applied Biosystems, USA).

**Expression Analysis**

Peripheral blood mononuclear cells were extracted from peripheral blood by density gradient centrifugation on Lymphodex. The MSCs were isolated and then incubated in Dulbecco’s Modified Eagle Medium/Nutrient Mixture F-12 supplemented with 10% fetal bovine serum. After reaching 70-80% confluence, these cells were differentiated into neuronal-like cells through culturing in the medium, including Noggin, as previously published (21). In this protocol, the bone morphogenesis pathway in MSCs was suppressed through Noggin treatment.

The neuronal-like cells obtained from the differentiation of PB-MSCs were used to investigate the expression of genes adjacent to the CGG repeat expansion of the *FMR1* gene (i.e., *SLITRK2*, *SLITRK4*, *GABRA3*, and *MECP2*) in females with full mutation and different presentation of clinical symptoms. The selected genes were located at Xq28 and Xq27. The previous studies indicated that these genes play an important role in the development of the nervous system. 

For this purpose, Total RNA was extracted from cell cultures using a Total RNA puriﬁcation kit (Jena Bioscience, Germany). Then, complementary deoxyribonucleic acid (cDNA) was synthesized by RevertAid First Strand cDNA Synthesis kit (Thermo Scientiﬁc, USA) with Random Hexamer Primers. Real-time PCR was performed according to the manufacturer’s protocol using RealQ Plus Master Mix Green (Ampliqon, Denmark). The expression of target genes, including *SLITRK2*, *SLITRK4*, *GABRA3*, and *MECP2*, was detected by the SYBR Green system and normalized with Heat Shock Protein 90 Alpha Family Class B Member 1, *HSP90AB1, *expression. The PCR primers were as follows: 

*SLITRK2*: 5-TGCAGTCATTCAGGAAGGTG-3 and 5-GCTCTGCAGTCCATCAAACA-3 (21) 

*SLITRK4*: 5-TCAGCCCTGATTTCTTCGACA-3 and 5-CTCACAGTTGACATAGAGCACAT-3 (22) 

*MECP2*: 5-TGAGATGCCTGGTGAGCATTACAG-3 and 5-TCCACCTTCCATACCACTCCCA-3 (23) 

*GABRA3*: 5-CATGAAGATCCTTCCACTGAACA-3 and 5-GGTTCCGTTGTCCACCAATC-3 (24) 

*HSP90AB1*: 5-GGAAGTGCACCATGGAGAGGA-3 and 5-GCGAATCTTGTCCAAGGCATCAG-3 

Then, the relative expression level of each gene was determined by the Pfaffl method (25). 

## Results

**Patient’s Characterization**

The present study was performed on a female with a normal allele and two females with *FMR1* full mutation. The females who were carriers of *FMR1* full mutation showed different phenotypes. One of them presented a normal phenotype; nevertheless, the other female showed the symptoms of FXS, including mental retardation, learning disability, autism, attention deficits, hyperactivity, attentional problems, poor eye contact, obsessional interests or behaviors, shyness or social anxiety, and prominent or large ears. 

**Assessment of** ***FMR1*** **Triplet Repeat Region**

The *TP PCR* followed by capillary electrophoresis analysis showed the presence of two normal alleles in the female control. In contrast, a full mutation allele was observed in the TP-PCR products of two other samples (NF and MF). AmplideX FMR1 PCR kit and Southern blot analysis also confirmed the presence of a normal allele and a full mutation allele in these two samples. The MS PCR of the *FMR1* triplet repeat region showed that the normal allele was un-methylated; nonetheless, the full mutation allele was methylated in the studied carrier females. 

**Expression Analysis** **of *FMR1* Flanking Genes**

**Figure 1:**
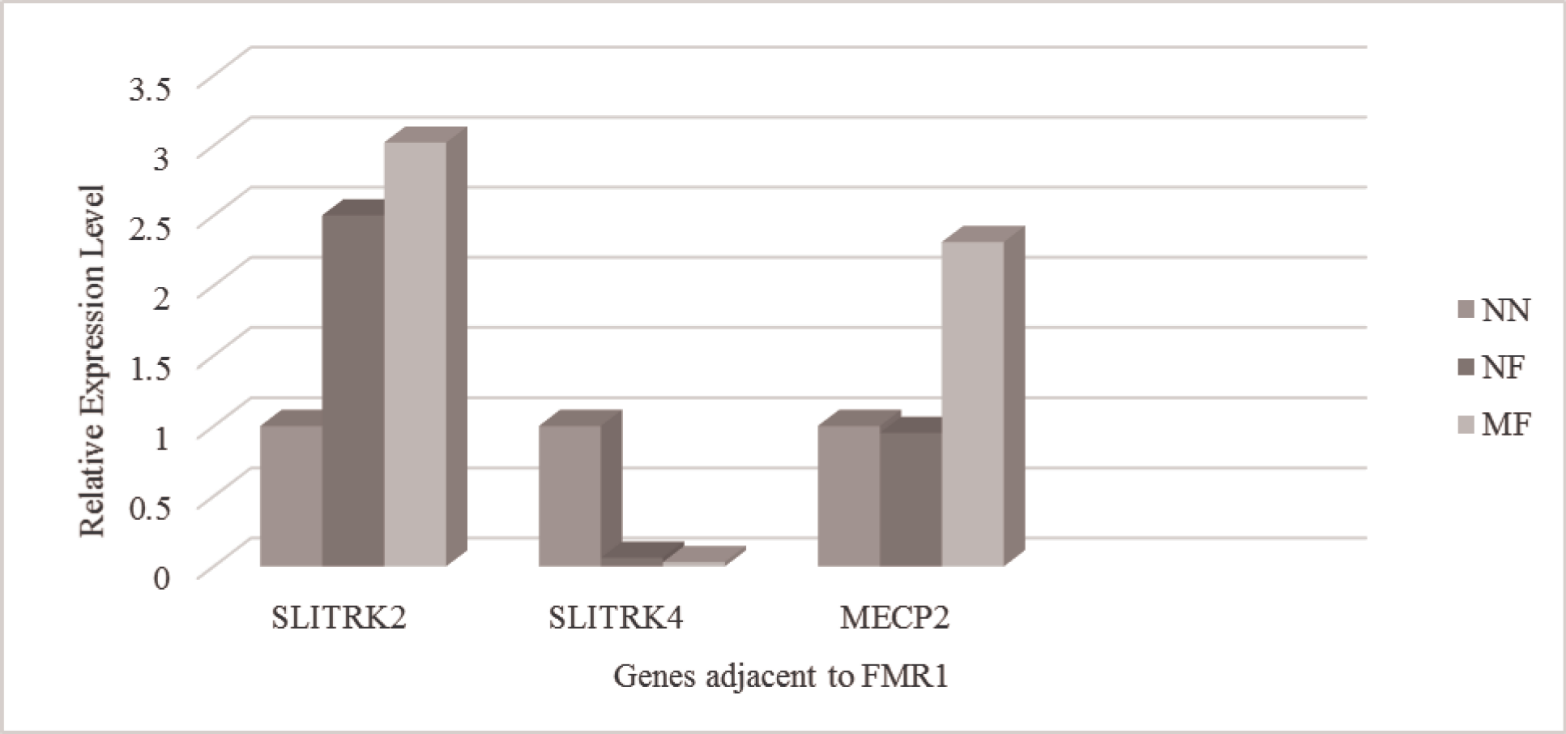
Expression Level of Genes Adjacent to the *FMR1* Gene, Including *SLITRK2*, *SLITRK4*, and *MECP2*, in the Neuronal-like Cells of Female Carriers of *FMR1* Full Mutation

**Figure 2:**
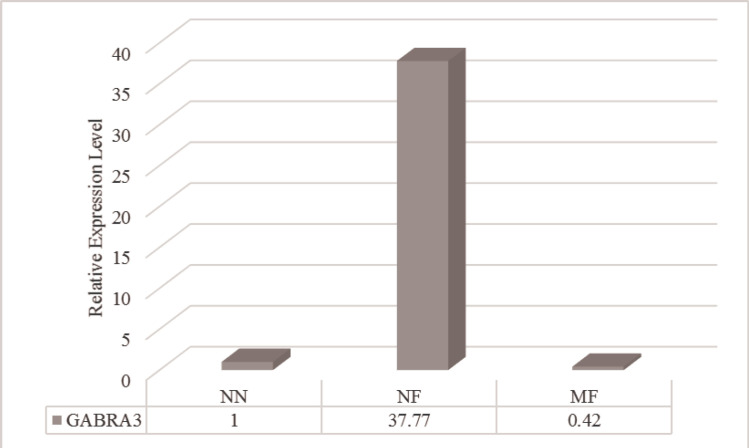
Expression Level of the *GABRA3* Gene Located Adjacent to Expansion of CGG Repeats of the *FMR1* Gene in the Neuronal-like Cells of Female Carriers of *FMR1* Full Mutation

The results obtained from real-time PCR indicated that the expression of *SLITRK2* upregulated in the neuronal cells differentiated from the PB-MSCs of females with the full mutation allele of the *FMR1* gene regardless of their phenotype. In contrast, the downregulation of *SLITRK4* expression was detected in these cells. No difference of *MECP2* expression was observed in the neuronal-like cells of the normal female with full mutation allele of the *FMR1* gene, compared to those of the female with the normal *FMR1* allele; nevertheless, *MECP2* expression increased in the neuronal-like cells of mental retardation female with the full mutation (Figure 1). Although the upregulation of the *GABRA3* gene was observed in the neuronal-like cells of the normal female with the full mutation allele, its expression was decreased in the neuronal-like cells of the mental retardation female with the full mutation of *FMR1* (Figure 2).

NF; Normal female with full mutation, MF; Mental retardation female with full mutation, NN; Normal female with normal allele of the *FMR1* gene

## Discussion

Among the heterogeneous group of neurodevelopmental disorders, FXS is an excellent model to study the molecular mechanisms of synaptic function that can cause cognitive impairment, autism, and behavioral disorders in the affected patients (25). The study of targeted therapies to correct synaptic changes can reveal the new treatment approach for not only FXS patients but also other types of neurodevelopmental diseases (26-27). In the present study, the relationship between the methylation patterns of CGG repeats located upstream of the *FMR1* gene and genes adjacent to *FMR1* was investigated in the neuron-like cells differentiated from the PB-MSCs of two females carrying *FMR1* full mutation with different phenotypes. 

Previous studies indicated that the abnormal methylation in the *FMR1* promoter region alleles containing CGG repeat expansion occurs in patients with FXS (28). The most severe phenotype of disease was observed in males that all alleles of this locus were methylated in all their cells, resulting in no expression of *FMR1* mRNA (29). Tassone et al. reported significant levels of *FMR1* mRNA expression in men with a methylated *FMR1* full mutation allele (30). However, no detectable levels of *FMR1* mRNA were noticed for some males with an unmethylated full mutation allele of *FMR1*. These individuals presented phenotypic symptoms similar to males with methylated full mutation allele (29). 

The results obtained from some studies suggested that there were three types of methylation mosaicism at the *FMR1* gene, including mosaicism among the cells of an individual, mosaicism at CpG sites within a gene, or mosaicism between the two strands of a single DNA molecule (29). In the present study, the abnormal allele of both females with full mutation was methylated; nonetheless, they showed different phenotypes. The methylation mosaicism could explain the observed phenotypic differences. These females with full mutation allele of the *FMR1* gene also showed different expression patterns of genes adjacent to the *FMR1* gene, compared to the normal female carrying the normal allele.

The expression analysis of *SLITRK2* and *SLITRK4* indicated that *SLITRK2* was overexpressed in the differentiated neuronal-like cells of females with *FMR1* full mutation; however, the expression of *SLITRK4* decreased in these cells, compared to those of the female with the normal allele. Marteyn et al. demonstrated that a stable knockdown of *SLITRK4* and *SLITRK2* genes in the neurons of wild-type cultures was associated with the induction of neurite formation (21). In contrast, the transient expression of *SLITRK2* and *SLITRK4* was accompanied by the inhibition of neurite outgrowth (31). The *SLITRK2* and *SLITRK4* were identified as the regulators of neurite outgrowth (32). Some studies suggested *SLITRK2 *as a candidate gene of bipolar disorder (33). Therefore, the upregulation of *SLITRK4*, along with the downregulation of *SLITRK2*, in the neuronal-like cells generated from females with *FMR1* full mutation could be proposed as the protective mechanism against the development of psychiatric disorders, including bipolar disorder, in individuals carrying the full mutation of *FMR1*. 

In the present study, the overexpression of the *MECP2* gene was observed in the neuronal-like cells differentiated from the PB-MSCs of mental retardation female carrying *FMR1* full mutation allele; nonetheless, *MECP2* gene expression showed no difference in the normal female with *FMR1* full mutation, compared to that of the female carrying the normal allele. The mental retardation phenotype observed, along with the increased expression of *MECP2*, is consistent with the previous observations. The study of *MECP2*-null mice indicated that loss of *MECP2 *damaged the learning and memory through the reduction of synaptic plasticity and spontaneous activity of neurons (34-36). Synaptic defects were also reported in transgenic mice that overexpressed *MECP2*, indicating that the regulation of *MECP2 *expression level is required for the maturation of neurons (37). The activation of immature neurons was dependent on the induction of a large number of genes contributing to the maturation of developing synapses. The *MECP2 *plays an important role in controlling the expression of these genes, including B*rain Derived Neurotrophic Factor* (BDNF)*, *Inhibitor of differentiation/DNA binding (ID1)*,* Early Growth Response 2 (EGR2)*, and *JUNB* (*38).

The expression of the *GABRA3* gene was also investigated in the present study. The results obtained from real-time PCR presented that the *GABRA3* gene was overexpressed in the neuronal-like cells of normal females with *FMR1* full mutation; however, the reduction of *GABRA3* expression was observed in the neuronal-like cells of the mental retardation female carrying the full mutation, compared to that of the normal female. 

Previous studies indicated that the lack of *FMRP* increased the degradation of gamma-aminobutyric acid (GABA) receptor subunits, leading to the electrophysiological and molecular defects of the GABAergic system (38-39). The level of the mRNA expression of seven GABA receptor subunits (i.e., α1, α3, α4, β1, β2, γ1, and γ2) reduced in the cortex of FMR1 knockout mice (39). Furthermore, the downregulation of the *GABRA3 *gene was observed in the cortex of patients with autism (40). Some studies revealed that dysfunction of GABA receptors is involved in the manifestation of clinical symptoms, including depression and problems with learning, memory, and behavioral phenotype of patients with FXS (41). The results of the current study about the downregulation of *GABRA3* gene expression in the neuronal-like cells obtained from the mental retardation female with full mutation allele are consistent with the results of previous studies.

The activation of GABA receptors containing subunits α2 and/or α3 has also been demonstrated to involve in the maturation and differentiation of neurons (42), and they showed high expression in the neuronal synapses of the brain areas affecting anxiety (43). Although other subunits of GABA receptors were not investigated, the observation of *GABRA3* (subunit α3) overexpression in the neuronal-like cells from the normal female with *FMR1* full mutation indicated that the upregulation of *GABRA3* plays a role as a compensatory mechanism in the expression changes of other GABA receptor subunits.

**In Conclusion**, the expression of *SLITRK2*, *SLITRK4*, *MECP2*, and *GABRA3* was observed to be associated with the phenotypic characteristics of females carrying the full mutation. The obtained results could play an important role in the understanding of novel therapeutic targets for patients with mild symptoms of FXS and patients affected by other FMR1-related disorders.

## Author’s contribution

MD. Omrani supervised the project; Z. Fazeli carried out the experiments and Wrote the manuscript; SMH. Ghaderian contributed to the interpretation of the results; H. Najmabadi helped in the collection of samples.

## Conflicts of Interest

The authors declare that they have no conflict of interest.

## References

[1] Martyn M, Anderson V, Archibald A, Carter R, Cohen J, Delatycki M (2013). Offering fragile X syndrome carrier screening: a prospective mixed-methods observational study comparing carrier screening of pregnant and non-pregnant women in the general population. BMJ Open..

[2] Crawford DC, Acuña JM, Sherman SL (2001). FMR1 and the fragile X syndrome: human genome epidemiology review. Genet Med..

[3] Hatton DD, Sideris J, Skinner M, Mankowski J, Bailey DB Jr, Roberts J (2006). Autistic behavior in children with fragile X syndrome: prevalence, stability, and the impact of FMRP. Am J Med Genet A..

[4] Moss J, Howlin P (2009). Autism spectrum disorders in genetic syndromes: implications for diagnosis, intervention and understanding the wider autism spectrum disorder population. J Intellect Disabil Res..

[5] Sherman S, Pletcher BA, Driscoll DA (2005). Fragile X syndrome: diagnostic and carrier testing. Genet Med..

[6] de Vries BB, Wiegers AM, Smits AP, Mohkamsing S, Duivenvoorden HJ, Fryns JP (1996). Mental status of females with an FMR1 gene full mutation. Am J Hum Genet..

[7] Munir F, Cornish KM, Wilding J (2000). Nature of the working memory deficit in fragile-X syndrome. Brain Cogn..

[8] Wilding J, Cornish K, Munir F (2002). Further delineation of the executive deficit in males with fragile-X syndrome. Neuropsychologia..

[9] Cornish KM, Munir F, Cross G (1999). Spatial cognition in males with Fragile-X syndrome: evidence for a neuropsychological phenotype. Cortex..

[10] Hall SS, Burns DD, Lightbody AA, Reiss AL (2008). Longitudinal changes in intellectual development in children with Fragile X syndrome. J Abnorm Child Psychol..

[11] Brouwer JR, Mientjes EJ, Bakker CE, Nieuwenhuizen IM, Severijnen LA, Van der Linde HC (2007). Elevated Fmr1 mRNA levels and reduced protein expression in a mouse model with an unmethylated Fragile X full mutation. Exp Cell Res..

[12] Tyson JA, Anderson SA (2013). The protracted maturation of human ESC-derived interneurons. Cell Cycle..

[13] Marín O (2013). Human cortical interneurons take their time. Cell Stem Cell..

[14] Gerhardt J (2015). Epigenetic modifications in human fragile X pluripotent stem cells; Implications in fragile X syndrome modeling. Brain Res..

[15] Kim S, Honmou O, Kato K, Nonaka T, Houkin K, Hamada H (2006). Neural differentiation potential of peripheral blood- and bone-marrow-derived precursor cells. Brain Res..

[16] Weiler IJ, Greenough WT (1999). Synaptic synthesis of the Fragile X protein: possible involvement in synapse maturation and elimination. Am J Med Genet..

[17] Weiler IJ, Irwin SA, Klintsova AY, Spencer CM, Brazelton AD, Miyashiro K (1997). Fragile X mental retardation protein is translated near synapses in response to neurotransmitter activation. Proc Natl Acad Sci U S A..

[18] Darnell JC, Richter JD (2012). Cytoplasmic RNA-binding proteins and the control of complex brain function. Cold Spring Harb Perspect Biol..

[19] Bittel DC, Kibiryeva N, Butler MG (2007). Whole genome microarray analysis of gene expression in subjects with fragile X syndrome. Genet Med..

[20] Zhou Y, Lum JM, Yeo GH, Kiing J, Tay SK, Chong SS (2006). Simplified molecular diagnosis of fragile X syndrome by fluorescent methylation-specific PCR and GeneScan analysis. Clin Chem..

[21] Marteyn A, Maury Y, Gauthier MM, Lecuyer C, Vernet R, Denis JA (2011). Mutant human embryonic stem cells reveal neurite and synapse formation defects in type 1 myotonic dystrophy. Cell Stem Cell..

[22] Squillaro T, Alessio N, Cipollaro M, Melone MA, Hayek G, Renieri A (2012). Reduced expression of MECP2 affects cell commitment and maintenance in neurons by triggering senescence: new perspective for Rett syndrome. Mol Biol Cell..

[23] Plummer PN, Colson NJ, Lewohl JM, MacKay RK, Fernandez F, Haupt LM (2011). Significant differences in gene expression of GABA receptors in peripheral blood leukocytes of migraineurs. Gene..

[24] Pfaffl MW (2001). A new mathematical model for relative quantification in real-time RT-PCR. Nucleic Acids Res..

[25] Castrén ML, Castrén E (2014). BDNF in fragile X syndrome. Neuropharmacology..

[26] Hagerman RJ, Berry-Kravis E, Kaufmann WE, Ono MY, Tartaglia N, Lachiewicz A (2009). Advances in the treatment of fragile X syndrome. Pediatrics..

[27] Healy A, Rush R, Ocain T (2011). Fragile X syndrome: an update on developing treatment modalities. ACS Chem Neurosci..

[28] Pieretti M, Zhang FP, Fu YH, Warren ST, Oostra BA, Caskey CT (1991). Absence of expression of the FMR-1 gene in fragile X syndrome. Cell..

[29] Stöger R, Genereux DP, Hagerman RJ, Hagerman PJ, Tassone F, Laird CD (2011). Testing the FMR1 promoter for mosaicism in DNA methylation among CpG sites, strands, and cells in FMR1-expressing males with fragile X syndrome. PLoS One..

[30] Tassone F, Hagerman RJ, Taylor AK, Hagerman PJ (2001). A majority of fragile X males with methylated, full mutation alleles have significant levels of FMR1 messenger RNA. J Med Genet..

[31] Aruga J, Mikoshiba K (2003). Identification and characterization of Slitrk, a novel neuronal transmembrane protein family controlling neurite outgrowth. Mol Cell Neurosci..

[32] Yim YS, Kwon Y, Nam J, Yoon HI, Lee K, Kim DG (2013). Slitrks control excitatory and inhibitory synapse formation with LAR receptor protein tyrosine phosphatases. Proc Natl Acad Sci U S A..

[33] Smith EN, Bloss CS, Badner JA, Barrett T, Belmonte PL, Berrettini W (2009). Genome-wide association study of bipolar disorder in European American and African American individuals. Mol Psychiatry..

[34] Moretti P, Levenson JM, Battaglia F, Atkinson R, Teague R, Antalffy B (2006). Learning and memory and synaptic plasticity are impaired in a mouse model of Rett syndrome. J Neurosci..

[35] Dani VS, Nelson SB (2009). Intact long-term potentiation but reduced connectivity between neocortical layer 5 pyramidal neurons in a mouse model of Rett syndrome. J Neurosci..

[36] Collins AL, Levenson JM, Vilaythong AP, Richman R, Armstrong DL, Noebels JL (2004). Mild overexpression of MeCP2 causes a progressive neurological disorder in mice. Hum Mol Genet..

[37] Gonzales ML, LaSalle JM (2010). The role of MeCP2 in brain development and neurodevelopmental disorders. Curr Psychiatry Rep..

[38] 38- D'Hulst C, De Geest N, Reeve SP, Van Dam D, De Deyn PP, Hassan BA (2006). Decreased expression of the GABAA receptor in fragile X syndrome. Brain Res..

[39] D'Hulst C, Heulens I, Brouwer JR, Willemsen R, De Geest N, Reeve SP (2009). Expression of the GABAergic system in animal models for fragile X syndrome and fragile X associated tremor/ataxia syndrome (FXTAS). Brain Res..

[40] Fatemi SH, Reutiman TJ, Folsom TD, Rustan OG, Rooney RJ, Thuras PD (2014). Downregulation of GABAA receptor protein subunits α6, β2, δ, ε, γ2, θ, and ρ2 in superior frontal cortex of subjects with autism. J Autism Dev Disord..

[41] Mihalek RM, Banerjee PK, Korpi ER, Quinlan JJ, Firestone LL, Mi ZP (1999). Attenuated sensitivity to neuroactive steroids in gamma-aminobutyrate type A receptor delta subunit knockout mice. Proc Natl Acad Sci U S A..

[42] Takayama C, Inoue Y (2004). Transient expression of GABAA receptor alpha2 and alpha3 subunits in differentiating cerebellar neurons. Brain Res Dev Brain Res..

[43] Geracitano R, Fischer D, Kasugai Y, Ferraguti F, Capogna M (2012). Functional expression of the GABA(A) receptor α2 and α3 subunits at synapses between intercalated medial paracapsular neurons of mouse amygdala. Front Neural Circuits..

